# Self-efficacy for cancer self-management in the context of COVID-19: a cross-sectional survey study

**DOI:** 10.1007/s00520-025-09905-9

**Published:** 2025-09-10

**Authors:** Thomas Lawler, Kristine Kwekkeboom, Shaneda Warren Andersen, Ajay K. Sethi, Amye J. Tevaarwerk, Kristin Litzelman, Priyanka A. Pophali, Ronald E. Gangnon, John M. Hampton, Noelle K. LoConte, Amy Trentham-Dietz

**Affiliations:** 1 Carbone Cancer Center, School of Medicine and Public Health, University of WI–Madison, Madison, WI USA; 2 School of Nursing, University of WI–Madison, Madison, WI USA; 3 Department of Population Health Sciences, School of Medicine and Public Health, University of WI–Madison, Madison, WI USA; 4 Department of Oncology, Mayo Clinic, Rochester, MN USA; 5 Mayo Clinic Comprehensive Cancer Center, Rochester, MN USA; 6 School of Human Ecology, University of WI–Madison, Madison, WI USA; 7 Division of Hematology, Medical Oncology and Palliative Care, Department of Medicine, School of Medicine and Public Health, University of WI–Madison, Madison, WI USA; 8 Department of Biostatistics and Medical Informatics, School of Medicine and Public Health, University of WI–Madison, Madison, WI USA

**Keywords:** Cancer, Neoplasms, Self-efficacy, Self-management, COVID-19

## Abstract

**Purpose:**

For cancer survivors, self-efficacy is needed to manage the disease and the effects of treatment. The COVID-19 pandemic disrupted cancer-related healthcare, which may have impacted self-management self-efficacy. We investigated self-efficacy reported by cancer survivors during COVID-19, including associations with healthcare disruptions, distress, and general health.

**Methods:**

Between 2020 and 2021, 1902 individuals aged 18–80 years with a recent cancer diagnosis completed a survey regarding the effects of COVID-19 on healthcare, self-efficacy for managing cancer and social interactions, cancer-related distress, and perceived general health. Linear and logistic models estimated odds ratios and 95% confidence intervals (CIs) between self-efficacy scores, healthcare disruptions, significant distress, and general health.

**Results:**

Mean self-efficacy for managing cancer was 7.58 out of 10. Greater self-efficacy was associated with lower odds for distress (OR 0.18 [95% CI 0.13–0.26], quartile 4 vs. 1) and for worse general health (0.05 [0.03–0.09]). Participants with disruptions to cancer-related healthcare had lower self-efficacy for managing cancer compared to those without (6.62 vs. 7.09, respectively, *P* < 0.001) and higher odds for distress (1.70 [1.36–2.14]), but not worse general health (1.13 [0.39–1.44]). Lower self-efficacy mediated 27% of the association between healthcare disruptions and increased distress (15–47%). Associations with self-efficacy for managing social interactions trended in the same direction.

**Conclusions:**

During COVID-19, disruptions to cancer-related healthcare were associated with lower self-efficacy, increased distress, and worse general health. Psychosocial interventions designed to overcome barriers and target self-efficacy may be important for enhancing outcomes among cancer survivors experiencing disruptions in healthcare access.

**Supplementary Information:**

The online version contains supplementary material available at 10.1007/s00520-025-09905-9.

## Introduction

Advances in cancer treatment have increased the number of people surviving a cancer diagnosis but have also increased the complexity and challenges of care [1, 2]. Side effects of new medications that extend survival and disease-related symptoms associated with cancer may require self-management by survivors and their families in their home settings [2, 3]. Self-management of cancer and its treatment, including associated symptoms, has become an important component of contemporary cancer care, with advocates citing the need for greater self-management support integrated throughout the cancer care trajectory [3].

The COVID-19 pandemic presented a number of challenges to cancer care delivery. Cancer survivors experienced significant disruptions in usual care, including fewer in-person visits with the cancer care team, the need to make clinic visits alone (without a caregiver or support person), and reduced access to both professional and lay supportive care providers [4]. These disruptions could have produced a negative impact on disease management in general and symptom management specifically. Moreover, initial evidence suggests that the stress and isolation of the COVID-19 pandemic resulted in a higher symptom burden for cancer survivors than documented pre-pandemic [5].

Bandura defined self-efficacy as an individual’s confidence in their ability to perform a specific task or affect a positive change in their life [6]. Self-efficacy is a critical component necessary to successfully engage in processes of cancer self-management. Conceptualized in a situation-specific manner, self-efficacy for managing cancer encompasses beliefs in one’s ability to control or cope with the effects of the disease and treatment. Prior research demonstrates that general and cancer-related self-efficacy is negatively associated with symptom severity and symptom distress and positively associated with general health and quality of life [7]. To our knowledge, no studies have reported self-efficacy for managing cancer and related symptoms during the height of COVID-19, including the impact of disruptions to cancer-related healthcare and associations with distress or perceived health. Altered opportunities for the care team to prepare cancer survivors and their families for cancer self-management due to COVID-19 restrictions and safety measures may have impacted survivors’ self-efficacy and contributed to cancer-related distress and diminished perceived health.

Therefore, we conducted a study to (1) describe self-management self-efficacy in cancer survivors during the COVID-19 pandemic; (2) identify demographic and clinical variables related to self-management self-efficacy, including disruptions to cancer-related healthcare during the pandemic; and (3) evaluate whether self-management self-efficacy mediates the association between disruptions to cancer-related healthcare and perceived general health and psychological well-being.

## Methods

### Participants and recruitment strategy

Eligible participants were adults (age 18–80 years at appointment) who completed a healthcare appointment at a University of Wisconsin Carbone Cancer Center (UWCCC) clinic between November 1, 2019, and June 30, 2020. All participants had an active diagnosis of malignant cancer first noted in the electronic health record (EHR) in 2019 or 2020. Further, eligible participants had a valid residential address and were identified via search of the UW’s EHR. Participants were eligible regardless of treatment history, cancer type or location, diagnostic stage, and time since initial diagnosis. There were 2961 cancer survivors invited to enroll in the study. Paper surveys were mailed to eligible participants, and follow-up mailings and phone calls to non-respondents were completed to maximize response and limit participation bias. Completed questionnaires were returned by mail. Participants were offered a $5 incentive for survey completion. In total, 1008 survivors declined to participate, including 24 who declined due to illness. Enrolled participants completed a survey between October 2020 and April 2021. Surveys were completed and returned by 1953 participants (66% of the eligible sample). Participants were excluded from this analysis who met any of the following criteria: (1) were diagnosed with non-melanoma skin cancer only (*N* = 11), (2) did not respond to the question asking whether they had ever been diagnosed with cancer (*N* = 7), or (3) did not respond to any of the questions concerning self-management self-efficacy (*N* = 33). In total, 1902 surveys were included in the final analysis. All participants provided informed consent, and all research activities conformed to the Declaration of Helsinki. The University of Wisconsin Health Sciences Institutional Review Board provided approval for this study.

### Survey design and evaluation of COVID-19 impacts on healthcare

Participants completed a questionnaire containing 80 questions over the following topics: (A) Cancer Diagnosis and Treatment, (B) COVID-19, (C) Impact of COVID-19 on Health and Healthcare, (D) General Health Status, (E) Lifestyle and Health Behaviors, (F) Other Impacts of COVID-19, and (G) Demographics. To capture social determinants of health, each participant’s residential ZIP code was categorized as previously described in the 2020 Wisconsin Health Disparities Report (rural, rural advantaged, rural disadvantaged, urban, urban advantaged, urban disadvantaged) [8]. In section (C), participants were queried about (1) whether COVID-19 had caused delays in receiving cancer-related care (adapted from Park et al. [9] to be specific to cancer-related care; yes, no); (2) whether COVID-19 had led to difficulties receiving cancer-related medications or other medications (yes, no); (3) whether COVID-19 had negatively impacted their ability to make decisions about cancer-related care; and (4) whether COVID-19 had negatively impacted their ability to obtain cancer-related care. Items 3 and 4 were evaluated using a Likert scale, with potential responses ranging from 1 (“not true of me at all”) to 7 (“very true of me”). A summary variable was created to identify all participants who responded affirmatively to at least one of the queries (i.e., participant responded “yes” to query 1 or 2 or ≥ 4 out of 7 for queries 3 or 4).

### Evaluation of self-efficacy for managing cancer and social interactions

For the purposes of this study, we conceptualized self-efficacy in the context of the COVID-19 pandemic to include self-efficacy for managing cancer as well as self-efficacy for managing social interactions. Individuals living with cancer during the pandemic faced a new need to navigate stay-at-home orders and distancing restrictions that may have negatively impacted their health-related social interactions. Self-efficacy for managing cancer, including symptoms, side effects, and other effects of the disease and treatment, was measured using the validated “Self-Efficacy for Managing Chronic Disease 6-Item Scale” [10, 11] modified to reflect cancer as the health condition. Self-efficacy for managing social interaction, including the ability to communicate with healthcare providers and other sources of emotional and instrumental support, was evaluated using the validated Patient-Reported Outcomes Measurement Information System (PROMIS) “self-efficacy for managing social interactions” short form [12]. All survey queries are listed in Table 1. For each participant, the mean self-efficacy for managing cancer was calculated by averaging across all six items in the scale. Likewise, the mean self-efficacy for managing social interactions was calculated by averaging across all eight items in the PROMIS instrument.

**Table 1:** Distribution of self-efficacy scores for 1902 participants with a history of cancer diagnosis

Self-efficacy for managing cancer (1–10^a^): Rate your confidence that you…	N	Mean (SD)	IQR
1) Can keep the fatigue caused by your cancer from interfering with things you want to do	1871	7.33 (2.37)	6–9
2) Can keep the physical discomfort or pain of your cancer from interfering with things you want to do	1849	7.74 (2.29)	6–10
3) Can keep the emotional distress caused by your cancer from interfering with things you want to do	1875	7.56 (2.25)	6–9
4) Can keep any other symptoms or health problems caused by your cancer from interfering with things you want to do	1865	7.22 (2.37)	6–9
5) Can do the different tasks/activities to manage your cancer to reduce your need to see a doctor	1847	7.94 (2.09)	7–10
6) Can do things other than taking medication to reduce how much your cancer affects your everyday life	1838	7.77 (2.21)	7–10
*Mean self-efficacy for managing cancer*	*1902*	*7.58 (1.99)*	*6–9*
Self-efficacy for managing social interactions (1–5^a^): Rate your confidence that you…			
1) Can talk about your health problems with someone	1881	4.50 (0.80)	4–5
2) Can find someone to take you to the doctor’s office	1874	4.67 (0.74)	5
3) Can get emotional support when you need it	1875	4.39 (0.93)	4–5
4) Can ask for help when you don’t understand something	1871	4.59 (0.73)	4–5
5) Have someone who helps you understand medical information	1830	4.50 (0.88)	4–5
6) Have someone to help you manage your daily activities if you need help	1845	4.33 (0.99)	4–5
7) Have someone to help you plan and make decisions related to your illness	1835	4.51 (0.90)	4–5
8) Can communicate well with your doctors and nurses	1875	4.57 (0.75)	4–5
*Mean self-efficacy for managing social interactions*	*1886*	*4.52 (0.67)*	*4–5*

^a^Higher scores reflect greater self-efficacy

### Evaluation of distress and general health status

Participants were asked to rate the level of distress they had experienced in the previous week using the National Comprehensive Cancer Network (NCCN) distress thermometer [13] on a scale from 0 (no distress) to 10 (extreme distress). A distress score of ≥ 4 was defined as “moderate to severe distress,” which is an accepted cut-point to identify clinically significant distress for individuals with cancer [14]. Perceived general health at the time of survey completion was evaluated using the SF-36 general health item [15]. The item uses a Likert scale, with potential responses including “poor,” “fair,” “good,” “very good,” and “excellent.”

### Statistical analysis

The distribution of all self-efficacy variables was determined by calculating the mean and standard deviation (SD), as well as the interquartile range (IQR). Linear regression modeling was used to estimate the associations between exposure variables and mean self-efficacy, including self-efficacy for managing cancer and self-efficacy for managing social interactions. Exposures of interest included demographic variables (age at diagnosis, sex, race, education, income, and urban/rural residence), cancer treatment status, impacts of the COVID-19 pandemic on cancer-related healthcare, general health status, and clinically significant distress. Model 1 was adjusted for age at survey completion (18–39, 40–59, 60–69, ≥ 70 years). Model 2 was further adjusted for demographic covariates that may influence self-efficacy, including sex (male, female), race (White, Black or African American, Asian or Pacific Islander, Hispanic, other/multiracial), education (high school graduate or less, some college, bachelor’s degree, graduate or professional degree), annual household income (< $20 k, $20–49 k, $50–99 k, ≥ $100 k), neighborhood-level socioeconomic status (rural, rural advantaged, rural underserved, urban, urban advantaged, urban underserved), month of diagnosis, time since initial diagnosis (< 1, 1–1.99, 2–2.99, and ≥ 3 years), cancer treatment status (on/off treatment), diagnosis with anxiety or depression (yes, no), and the impact of the COVID-19 pandemic on depression (1–7). To conserve space, only results from Model 2 are included in the manuscript. Results from Model 1 are presented in Supplementary Tables S1-S3.

Logistic regression modeling was used to estimate odds ratios (ORs) with 95% confidence intervals (CIs) for clinically significant distress, defined as scores ≥ 4 out of 10 (vs. 1–3) on the National Comprehensive Cancer Network distress thermometer [13], and poor/fair overall health (vs. good/very good/excellent) by quartile of self-efficacy, per one unit increase in self-efficacy, and for participants who experienced negative impacts of the COVID-19 pandemic on cancer-related healthcare. Adjustment for covariates was completed as described above. To determine whether self-efficacy mediated the association between negative healthcare impacts of COVID-19 and significant distress, a formal mediation analysis was conducted using the *mediation* package in R (version 4.5.0) [16]. In brief, the *mediation* package estimates the direct and indirect effects of an exposure on an outcome of interest using two models: (1) a model for the mediating variable including the exposure of interest and all covariates and (2) a model for the outcome of interest including the exposure of interest, the mediating variable, and all other covariates [16]. Ninety-five percent CIs for the direct and indirect effects were estimated via bootstrapping with 1000 simulations.

Because missing values for covariates were uncommon (approximately 1–2% of observations), missing values were imputed using the age and sex-specific mode value. All statistical analyses were completed using SAS version 9.4 (SAS Inc., Cary, NC), except for the mediation analysis, which used R (version 4.5.0).

## Results

### Participant characteristics and self-efficacy levels

In total, 1902 participants with a previous cancer diagnosis were included in the analysis. Participants predominantly reported non-Hispanic White racial identity (95%), while a majority were female (58%). Seventy-six percent of participants reported annual household incomes ≥ $50,000 per year. Forty-three percent self-reported actively receiving a cancer treatment at the time of survey completion (inclusive of hormonal therapies). Most participants reported a high level of self-efficacy for managing cancer (mean: 7.6 out of 10 [SD: 2.0]). Scores were similar across all six items, including items targeting symptom management and those targeting disease management (Table 1). Likewise, participants reported high self-efficacy for managing social interactions (mean: 4.5 out of 5 [SD: 0.7]), with similar scores across all eight items (Table 1).

Higher self-efficacy for managing cancer and associated symptoms was observed for participants with female sex, higher education level, and income (Table 2, Supplementary Table S1). Participants who reported “Black/African American,” “Asian,” or “other/multiracial” identity had lower self-efficacy for managing cancer compared to White or Hispanic participants. Participants currently receiving treatment for cancer had lower self-efficacy for managing cancer compared to participants who were off treatment (mean [SE]: 6.67 [0.17] vs. 7.30 [0.17], respectively). Self-efficacy for managing cancer was also lower for participants diagnosed with cancer in the previous year and those with a history of depression or anxiety. Self-efficacy for managing social interactions was greater with higher income and lower among participants with Black/African American identity, but it did not vary substantially according to age, sex, educational level, or treatment status (Table 2, Supplementary Table S1).

**Table 2:** Sample characteristics and mean self-efficacy by category of demographic and treatment variables

	Sample characteristics (*N* = 1902)	Self-efficacy for managing cancer		Self-efficacy for managing social interactions	
	*N* (%)	*N*	Mean (SE)	*N*	Mean (SE)
			Model 2^d^		Model 2^d^
Age at diagnosis (years)					
18–39	99 (5.21%)	99	7.04 (0.24)	99	4.33 (0.08)
40–59	550 (28.92%)	550	6.85 (0.17)	547	4.34 (0.06)
60–69	712 (37.43%)	712	7.03 (0.17)	705	4.33 (0.06)
70 +	541 (28.44%)	541	7.00 (0.18)	535	4.30 (0.06)
Sex					
Male	807 (42.43%)	807	6.82 (0.17)	801	4.33 (0.06)
Female	1095 (57.57%)	1095	7.15 (0.17)	1085	4.32 (0.06)
Race/ethnicity					
White	1793 (94.27%)	1793	7.40 (0.10)	1777	4.43 (0.04)
Black/African American	31 (1.63%)	31	6.42 (0.34)	31	4.10 (0.12)
Asian/Pacific Islander	22 (1.16%)	22	6.81 (0.40)	22	4.30 (0.14)
Hispanic	29 (1.52%)	29	7.44 (0.35)	29	4.42 (0.12)
Other or multiracial	27 (1.42%)	27	6.83 (0.36)	27	4.37 (0.13)
Education					
High school or less	423 (22.24%)	423	6.84 (0.18)	419	4.29 (0.06)
Some college	635 (33.39%)	635	6.82 (0.17)	627	4.30 (0.06)
Bachelor’s degree	435 (22.87%)	435	7.19 (0.19)	433	4.37 (0.07)
Graduate or professional degree	409 (21.5%)	409	7.08 (0.19)	407	4.33 (0.07)
Annual household income					
< $20,000	115 (6.05%)	115	6.82 (0.23)	115	4.13 (0.08)
$20,000–$49,999	341 (17.93%)	341	6.90 (0.18)	339	4.27 (0.06)
$50,000–$99,999	854 (44.90%)	854	6.97 (0.17)	844	4.40 (0.06)
≥ $100,000	592 (31.13%)	592	7.25 (0.18)	588	4.50 (0.06)
Urban/rural residence					
Rural	374 (19.66%)	374	6.85 (0.18)	372	4.31 (0.06)
Rural advantaged	202 (10.62%)	202	7.06 (0.20)	199	4.36 (0.07)
Rural underserved	67 (3.52%)	67	6.88 (0.27)	67	4.34 (0.10)
Urban	247 (12.99%)	247	6.86 (0.19)	245	4.36 (0.07)
Urban advantaged	957 (50.32%)	957	7.11 (0.16)	950	4.30 (0.06)
Urban underserved	55 (2.89%)	55	7.13 (0.28)	53	4.29 (0.10)
Treatment status					
Off treatment	1077 (56.62%)	1077	7.30 (0.17)	1070	4.32 (0.06)
On treatment	825 (43.38%)	825	6.67 (0.17)	816	4.33 (0.06)
Most recent cancer diagnosis					
Breast	369 (19.87%)	369	6.93 (0.18)	365	4.34 (0.06)
Hematological cancers^a^	78 (4.20%)	78	6.69 (0.26)	74	4.22 (0.09)
Female reproductive cancers^b^	105 (5.65%)	105	6.75 (0.23)	104	4.22 (0.08)
Prostate	241 (12.98%)	241	7.18 (0.20)	241	4.25 (0.07)
Lung	256 (13.79%)	256	7.25 (0.21)	255	4.37 (0.07)
Colorectal	363 (19.55%)	363	6.84 (0.18)	363	4.39 (0.06)
Other^c^	445 (23.96%)	445	7.60 (0.19)	443	4.32 (0.07)
Years since cancer diagnosis					
< 1	353 (18.56%)	353	6.72 (0.18)	350	4.33 (0.06)
1–1.99	1061 (55.78%)	1061	6.97 (0.17)	1054	4.33 (0.06)
2–2.99	304 (15.98%)	304	7.15 (0.19)	301	4.34 (0.07)
≥ 3	184 (9.67%)	184	7.09 (0.20)	181	4.30 (0.07)
Depression or anxiety diagnosis					
No	1338 (70.35%)	1338	7.11 (0.17)	1323	4.35 (0.06)
Yes	564 (29.65%)	564	6.69 (0.18)	563	4.27 (0.06)

*SE* standard error

^a^Hematological cancers include leukemia and lymphoma

^b^Female reproductive cancers include vulvar, vaginal, cervical, endometrial, uterine, ovarian, and fallopian

^c^Other cancer types with less than 50 survey responses including kidney, melanoma, and others

^d^Adjusted for age at survey completion, race, sex, education, income, urban/rural advantage, treatment status, month of survey completion, years since initial cancer diagnosis, history of depression or anxiety, and the impact of COVID-19 on depression

### Self-efficacy and effects of COVID-19 on cancer-related care

Six hundred eighty-two participants (37%) reported that they experienced at least one type of negative impact of the COVID-19 pandemic on cancer-related healthcare (Table 3). For participants who reported any negative impact of COVID-19 on cancer care, self-efficacy for managing cancer was lower compared to participants who did not (mean [SE]: 6.62 [0.17] vs. 7.09 [0.17], respectively). Lower self-efficacy for managing cancer and associated symptoms was also associated with problems getting prescription medications during the COVID-19 pandemic, delays in cancer care, and negative impacts on the ability to make healthcare decisions or obtain cancer-related care (Table 3). Similar associations were observed for self-efficacy for managing social interactions (Table 3, Supplementary Table S2).

**Table 3:** Mean self-efficacy by whether participants experienced interruptions to cancer-related healthcare during the COVID-19 pandemic (*N* = 1898)

	Self-efficacy for managing cancer		Self-efficacy for managing social interactions	
	*N*	Mean (SE)	*N*	Mean (SE)
		Model 2^c^		Model 2^c^
Overall negative impacts of COVID-19 on cancer care^a^				
No	1169	7.09 (0.17)	1161	4.40 (0.06)
Yes	682	6.62 (0.17)	676	4.29 (0.06)
* P*-value^b^		< *.001*		< *.001*
Self-reported problems getting specific prescription medications because of COVID-19				
No	1818	6.99 (0.16)	1805	4.34 (0.06)
Yes	67	6.31 (0.27)	66	4.12 (0.09)
* P*-value^b^		*.003*		*.006*
Self-reported a delay in cancer care because of COVID-19				
No	1330	7.06 (0.17)	1319	4.42 (0.06)
Yes	522	6.74 (0.18)	518	4.28 (0.06)
* P*-value^b^		< *.001*		*.008*
COVID-19 negatively impacted the ability to make decisions about healthcare				
1 (not true of me at all)	1218	7.30 (0.16)	1210	4.42 (0.06)
2–3	419	6.58 (0.18)	417	4.18 (0.06)
4–5	155	6.19 (0.21)	154	4.15 (0.08)
6–7 (very true of me)	92	6.19 (0.24)	89	4.18 (0.09)
* P*-value^b^		< *.001*		< *.001*
COVID-19 negatively impacted the ability to obtain cancer care				
1 (not true of me at all)	1357	7.25 (0.16)	1347	4.43 (0.06)
2–3	320	6.60 (0.18)	317	4.21 (0.06)
4–5	125	6.13 (0.22)	124	4.21 (0.07)
6–7 (very true of me)	83	6.25 (0.25)	83	4.06 (0.08)
* P*-value^b^		< *.001*		< *.001*

*SE* standard error

^a^Participant responded “yes” to experiencing delays in receiving cancer-related care or problems receiving essential prescription medications or expressed agreement (score ≥ 4 out of 7) with the statement “The COVID-19 epidemic has negatively impacted my ability to make decisions about my cancer care” or “The COVID-19 epidemic has negatively impacted my ability to obtain cancer care”

^b^*P*-values represent a test of the null hypothesis that the mean values are equal across all groups

^c^Estimates adjusted for age at survey completion, race, sex, education, income, urban/rural advantage, treatment status, month of survey completion, years since initial cancer diagnosis, history of depression or anxiety, and the impact of COVID-19 on depression

### Self-efficacy, healthcare effects of COVID-19, and health outcomes

Forty-three percent of participants reported experiencing moderate to severe distress in the previous week, and 24% reported overall poor/fair general health. Participants who reported any negative impacts of COVID-19 on cancer-related healthcare were more likely to report clinically significant distress compared to participants who did not (OR 1.70, 95% CI: 1.36–2.14, Table 4). No association was observed for poor/fair general health (OR 1.13, 95% CI: 0.89–1.44). Participants in the highest quartile of self-efficacy for managing cancer and associated symptoms were less likely to report clinically significant distress (OR 0.18, 95% CI: 0.13–0.26, quartile 4 vs. 1) or poor/fair general health (OR 0.05, 95% CI: 0.03–0.09) compared to participants in the lowest quartile. Similar associations were observed for self-efficacy for managing social interactions (Table 4, Supplementary Table S3).

**Table 4:** Odds ratios (with 95% confidence intervals) for clinically significant distress and poor/fair general health for participants who experienced a disruption in cancer-related care due to COVID-19 and by level of self-efficacy (*N* = 1897)

	Significant distress^d^		Poor/fair general health	
	Yes/total	OR (95% CI)	Yes/total	OR (95% CI)
		Model 2^e^		Model 2^e^
Overall negative impacts of COVID-19 on cancer care^a^				
No	421/1161	1 (ref)	253/1153	1 (ref)
Yes	369/672	1.70 (1.36–2.14)	183/665	1.13 (0.89–1.44)
* P-*value^b^		< *.001*		*.31*
Self-reported problems getting specific prescription medications because of COVID-19				
No	762/1801	1 (ref)	414/1785	1 (ref)
Yes	42/66	1.84 (1.03–3.29)	30/66	2.12 (1.22–3.68)
* P*-value^b^		*.04*		*.007*
Self-reported a delay in cancer care because of COVID-19				
No	521/1319	1 (ref)	309/1309	1 (ref)
Yes	265/514	1.45 (1.14–1.85)	123/507	0.96 (0.74–1.24)
* P*-value^b^		*.03*		*.75*
COVID-19 negatively impacted the ability to make decisions about healthcare				
1 (not true of me at all)	399/1208	1 (ref)	225/1198	1 (ref)
2–3	232/413	1.66 (1.28–2.16)	132/412	1.86 (1.41–2.45)
4–5	116/154	3.63 (2.34–5.63)	57/151	2.00 (1.35–2.97)
6–7 (very true of me)	54/91	1.54 (0.90–2.64)	30/90	1.25 (0.74–2.10)
* P*-value^b^		< *.001*		< *.001*
COVID-19 negatively impacted the ability to obtain cancer care				
1 (not true of me at all)	484/1347	1 (ref)	272/1337	1 (ref)
2–3	188/315	1.74 (1.30–2.32)	101/313	1.67 (1.24–2.24)
4–5	83/124	1.99 (1.26–3.12)	47/121	1.90 (1.24–2.91)
6–7 (very true of me)	48/82	1.75 (1.02–3.02)	26/80	1.31 (0.76–2.24)
* P*-value^b^		<.001		< *.001*
General health				
Excellent	25/11	1 (ref)	–	–
Very good	161/548	1.33 (0.77–2.29)	–	–
Good	302/743	1.97 (1.16–3.35)	–	–
Fair	233/365	4.35 (2.47–7.65)	–	–
Poor	67/77	19.43 (8.04–46.94)	–	–
* P*-value^b^		< *.001*	*–*	*–*
Mean self-efficacy for managing cancer^c^				
Q1 (1.0–6.3)	333/469	1 (ref)	263/461	1 (ref)
Q2 (6.4–7.8)	220/431	0.51 (0.37–0.69)	102/430	0.24 (0.18–0.33)
Q3 (8.0–9.0)	153/499	0.24 (0.17–0.33)	57/494	0.11 (0.08–0.16)
Q4 (9.2–10.0)	102/481	0.18 (0.13–0.26)	26/481	0.05 (0.03–0.09)
* P*-value^b^		< *.001*		< *.001*
Continuous (per 1 unit increase)		0.71 (0.67–0.76)		0.57 (0.53–0.61)
Mean self-efficacy for managing social interactions^c^				
Q1 (1.0–2.9)	51/63	1 (ref)	36/61	1 (ref)
Q2 (3.0–3.9)	144/223	0.34 (0.16–0.74)	74/223	0.36 (0.19–0.67)
Q3 (4.0–4.9)	383/815	0.24 (0.12–0.50)	206/802	0.32 (0.18–0.58)
Q4 (5)	227/768	0.14 (0.06–0.28)	127/767	0.23 (0.13–0.41)
* P*-value^b^		< *.001*		< *.001*
Continuous (per 1 unit increase)		0.52 (0.44–0.63)		0.66 (0.56–0.78)

^a^Participant responded “yes” to experiencing delays in receiving cancer-related care or problems receiving essential prescription medications or expressed agreement (score ≥ 4 out of 7) with the statement “The COVID-19 epidemic has negatively impacted my ability to make decisions about my cancer care” or “The COVID-19 epidemic has negatively impacted my ability to obtain cancer care”

^b^*P*-values represent a test of the null hypothesis that the mean values are equal across all groups

^c^Higher scores reflect greater self-efficacy

^d^Defined as a score ≥ 4 out of 10 on the National Comprehensive Cancer Network distress thermometer

^e^Estimates adjusted for age at survey completion, race, sex, education, income, urban/rural advantage, treatment status, month of survey completion, years since initial cancer diagnosis, history of depression or anxiety, and the impact of COVID-19 on depression

In mediation analysis, lower self-efficacy for managing cancer partially mediated the association between negative healthcare impacts of COVID-19 and clinically significant distress, explaining 27% (95% CI: 15–47%) of the association (Table 5). Lower self-efficacy for managing social interactions mediated 15% of the association between healthcare impacts of COVID-19 and clinically significant distress (95% CI: 7–28%).

**Table 5:** Odds ratios (with 95% confidence intervals) for negative health outcomes among individuals who experienced interruptions to cancer-related healthcare because of COVID-19, including mediation by self-efficacy

Negative effects of the COVID-19 pandemic on cancer healthcare^a^	Significant distress^c^		
	OR (95% CI)^d^	Proportion mediated (%)	*P*-value^e^
No	1 (ref)	–	
Yes	1.70 (1.36–2.13)	–	
Yes—adjusted for self-efficacy for managing cancer^b^	1.52 (1.20–1.93)	27 (15–47)%	<.001
Yes—adjusted for self-efficacy for managing social interactions	1.60 (1.27–2.02)	15 (7–28)%	<.001

*CI* confidence interval, *OR* odds ratio

^a^Participant responded “yes” to experiencing delays in receiving cancer-related care or problems receiving essential prescription medications or expressed agreement (score ≥ 4 out of 7) with the statement “The COVID-19 epidemic has negatively impacted my ability to make decisions about my cancer care” or “The COVID-19 epidemic has negatively impacted my ability to obtain cancer care”

^b^Represents the mean of all six symptom management self-efficacy questions (see Table 1)

^c^Defined as a score ≥ 4 out of 10 on the National Comprehensive Cancer Network distress thermometer

^d^Estimates adjusted for age at survey completion, race, sex, education, income, urban/rural advantage, treatment status, month of survey completion, years since initial cancer diagnosis, history of depression or anxiety, and the impact of COVID-19 on depression

^e^*P*-values represent a test of the null hypothesis that the proportion mediated is equal to 0%

## Discussion

Sufficient self-efficacy is needed for an individual to implement behavioral changes to manage or improve their health [7]. In the domain of managing cancer treatment and symptoms, higher self-efficacy has been shown to be associated with lower symptom severity [17–19], less depression and anxiety [20, 21], and higher quality of life [7, 17, 22, 23]. However, disruptions to cancer-related healthcare during the COVID-19 pandemic increased the need for effective cancer self-management in the home setting [3] but may have negatively affected survivors’ self-efficacy for performing this [4]. Consequently, we surveyed cancer survivors about their self-efficacy during the pandemic and investigated associations with clinical and demographic factors, disruptions to healthcare, and self-reported health outcomes, including distress and perceived overall health.

Participants reported high self-efficacy for managing cancer, suggesting that most individuals felt confident in their ability to manage their cancer-related fatigue, physical discomfort, emotional distress, and other symptoms. Although we were not able to evaluate self-efficacy both before and after the onset of the pandemic, self-efficacy for managing cancer was similar to a comparable sample of US adults managing chronic disease assessed prior to COVID-19 [12]. Participants also reported high self-efficacy for managing social interactions, reflecting confidence in their ability to obtain emotional support and help with daily activities and to communicate with doctors and nurses. Higher self-efficacy for managing cancer was associated with higher educational attainment and income, suggesting that survivors with greater financial resources, better insurance coverage, or more confidence navigating the healthcare system feel better equipped to manage their condition. Consistent with a previous study of breast cancer survivors by Mosher et al. [24], we also found that self-efficacy for managing cancer was lower for participants who reported Black or African American race, which may reflect differences in wealth or other socioeconomic factors. Self-efficacy for managing cancer was higher for women, which conflicts with previous studies of cancer survivors showing lower self-efficacy in women or no association with gender [20, 25, 26]. An association was also observed for lower self-efficacy for managing cancer among participants diagnosed with depression or anxiety [21, 27, 28], highlighting the role of mental health in supporting cancer self-management. Notably, self-efficacy for managing cancer was also lower for participants currently receiving treatment for cancer or who were diagnosed within the previous year, consistent with previous studies demonstrating that self-efficacy is lower for individuals who are managing symptoms [18, 19]. This finding may also indicate that survivors who were newly diagnosed or recently started on cancer treatment felt overwhelmed and were more significantly affected than cancer survivors who had been managing treatment and symptoms for longer.

As previously reviewed, disruptions to cancer-related healthcare during the COVID-19 pandemic had the potential to exacerbate pre-existing psychological distress among vulnerable cancer survivors [29]. Notably, we found that participants with higher self-efficacy, including self-efficacy for managing cancer and for managing social interactions, were less likely to report clinically significant distress in the previous week. This is consistent with previous studies [7, 24, 30, 31] and indicates that cancer survivors who are confident in their ability to (1) obtain emotional support and (2) prevent cancer-related fatigue/symptoms from affecting their lives are less likely to experience mental health outcomes that negatively impact quality of life. Distress has been defined as “a multifactorial, unpleasant experience of a psychologic (i.e., cognitive, behavioral, emotional), social, spiritual, and/or physical nature that may interfere with the ability to cope effectively with cancer, its physical symptoms, and its treatment” and affects approximately 20–52% of cancer survivors [32]. Survivors experiencing distress are at higher risk for non-adherence to cancer treatment [33] and may have greater difficulty making decisions about treatment, requiring more frequent visits to the physician’s office and emergency department [34]. Distress is also associated with lower quality of life and increased mortality risk [33, 35]. Clinical trials have shown that interventions that include education, goal setting, and social support are effective for increasing self-efficacy and reducing symptom-related distress among individuals with cancer [30, 36, 37].

We also found that participants with higher self-efficacy (for managing cancer and social interactions) were less likely to report perceived poor/fair general health, consistent with prior research [18]. This may reflect these participants’ ability to manage cancer-related emotional distress, fatigue, and symptoms that negatively affect quality of life and perceived health, or their ability to engage healthcare providers or family members to help manage their treatment. However, this association may also indicate that participants with fewer cancer-related complications and good general health felt more confident in their ability to manage their symptoms.

Notably, average self-efficacy for managing cancer and social interactions was lower for participants who experienced disruptions to cancer-related healthcare during the pandemic, including difficulty receiving treatment, obtaining medications, or making healthcare decisions. The COVID-19 pandemic created significant challenges for the treatment and management of cancer [4, 38]. In part, delays and interruptions in treatment were caused by the burden of COVID-19 on healthcare resources and providers [4]. Further, during the pandemic, many cancer survivors were immunocompromised and vulnerable to severe COVID-19 infection requiring mechanical ventilation [39], making it necessary for providers and survivors to weigh the risks and benefits of cancer treatment [40]. To this end, multiple professional organizations issued guidelines to help providers and identify those whose treatment could safely be delayed or modified to reduce the risk for COVID-19 [41]. Evidence suggests that treatment delays contributed to poor disease control and increased risk for mortality among affected individuals, as previously reviewed [42]. By preventing individuals from receiving treatment or medications required to control symptoms or prevent disease progression, healthcare disruptions may have negatively influenced individuals’ self-efficacy for managing their cancer, leading to feelings of distress and anxiety. Consistent with this hypothesis, we observed that survivors who reported pandemic-related healthcare disruptions were more likely to have experienced distress in the previous week and that this association was partially mediated by self-efficacy. However, alternative explanations for our findings are also possible, as individuals with lower self-efficacy may have been more likely to perceive disruptions to healthcare or to have difficulty navigating potential pandemic-related changes, leading to greater disruption.

In the present analysis, we found that self-efficacy (including for cancer self-management and for managing social interactions) was related to demographic background and variables reflecting disruptions to healthcare during the COVID-19 pandemic, and that self-efficacy may mediate the associations between healthcare disruptions and self-reported distress. While these findings were cross-sectional and do not demonstrate causality, the results do suggest that providing additional support to cancer survivors with lower self-efficacy may modify the impacts of external healthcare disruptions and lead to improvements in distress. Importantly, psycho-oncologist-led and web-based interventions are effective for increasing self-efficacy in cancer survivors [43, 44] and should be prioritized among persons who may have short- or long-term delayed access to care. Findings of lower self-efficacy among low-income participants and those from minority racial groups, populations that were disproportionately impacted by the COVID-19 pandemic [45], highlight the importance of continued efforts to address health disparities, including disparities in self-efficacy. There is an ongoing need for development and testing of cancer self-efficacy interventions tailored to the unique needs of these marginalized populations. Such efforts could mitigate the impact of current and future disruptions in care.

This study has several limitations. Primarily, we utilized a cross-sectional study design and consequently are not able to identify causal relationships among the variables of interest or determine whether self-efficacy for managing cancer changed during the COVID-19 pandemic. The cross-sectional design also limits the interpretation of models for mediation analysis, as a temporal relationship between the variables cannot be established. Secondly, it is possible that participants with more severe symptoms had lower self-efficacy, which may have influenced associations with other variables. Unfortunately, we did not include questions related to symptom distress in the study survey. However, we attempted to control for the effect of symptoms by adjusting for whether participants were on or off treatment. Further, our measure of emotional distress was not specific to cancer and consequently may have been impacted by stressors related to other aspects of healthcare or the pandemic. Lastly, people in our sample were relatively affluent with predominantly non-Hispanic White racial identity, reflecting the demographic characteristics of our cancer center’s catchment area. Consequently, the results may not generalize to more diverse populations. Strengths of our study include the inclusion of both urban and rural residents as well as the timing of the survey, which queried a large sample of cancer survivors while many pandemic-related restrictions were still in place.

## Conclusions

In conclusion, we observed relatively high self-efficacy for disease management among cancer survivors receiving care from the University of Wisconsin Carbone Cancer Center during the COVID-19 pandemic despite unprecedented interruptions to cancer-related healthcare. Further, the present results demonstrate clear associations between higher self-efficacy for cancer management and lower levels of distress and perceived poor health in the context of a global pandemic, as well as a link between interruptions to cancer-related healthcare and lower self-efficacy. Well-designed interventions to overcome challenges in healthcare access and augment self-efficacy (e.g., telephone, local healthcare partners, and culturally matched peer survivors) may help to mitigate the effects of future healthcare disruptions on self-reported health outcomes, including distress.

## Supplementary Information

Below is the link to the electronic supplementary material.

ESM 1 (DOCX 41.5 KB)

## Data Availability

The data that support the findings of this study are not openly available due to reasons of sensitivity and are available from the corresponding author upon reasonable request.
