# Supplementary material for: Self-efficacy for cancer self-management in the context of COVID-19: a cross-sectional survey study
Source: Support Care Cancer. 2025 Sep 10;33(10):843. doi: 10.1007/s00520-025-09905-9 (PMC12423133; doi:10.1007/s00520-025-09905-9)
Supplement: Supplementary file 1 — (DOCX 41.5 KB) [file 520_2025_9905_MOESM1_ESM.docx]

**Title:** Self-efficacy for cancer self-management in the context of COVID-19, a cross-sectional survey study

**Journal:** Supportive care in cancer

**Author names:** Tom Lawler, PhD, Kristine Kwekkeboom, PhD, RN, FAAN, Shaneda Warren Andersen, PhD, Ajay K. Sethi, PhD, MHS, Amye J. Tevaarwerk, MD, Kristin Litzelman, PhD, Priyanka A. Pophali, MD, Ronald E. Gangnon, PhD, John M. Hampton, MS, Noelle K. LoConte, MD, FASCO, Amy Trentham-Dietz, PhD

**Corresponding author:**

Tom Lawler, PhD

Carbone Cancer Center, University of Wisconsin – Madison

Phone: 608-265-9606

Email: [tlawler2@wisc.edu](mailto:tlawler2@wisc.edu)

| **Supplementary Table S1:** Sample characteristics and mean self-efficacy by category of demographic and treatment variables | | | | | | | |
| --- | --- | --- | --- | --- | --- | --- | --- |
|  | **Sample characteristics (N = 1,902)** | **Mean self-efficacy**  **for managing cancer** | | | **Mean self-efficacy**  **for managing social interactions** | | |
|  | **N (%)** | **N** | **Mean (SE)**  **Model 1**^a^ | **Mean (SE)**  **Model 2**^a^ | **N** | **Mean (SE)**  **Model 1**^a^ | **Mean (SE)**  **Model 2**^a^ |
| **Age at diagnosis (years)** |  |  |  |  |  |  |  |
| 18-39 | 99 (5.21%) | 99 | 7.52 (0.20) | 7.04 (0.24) | 99 | 4.48 (0.07) | 4.33 (0.08) |
| 40-59 | 550 (28.92%) | 550 | 7.47 (0.08) | 6.85 (0.17) | 547 | 4.53 (0.03) | 4.34 (0.06) |
| 60-69 | 712 (37.43%) | 712 | 7.59 (0.07) | 7.03 (0.17) | 705 | 4.52 (0.03) | 4.33 (0.06) |
| 70+ | 541 (28.44%) | 541 | 7.69 (0.09) | 7.00 (0.18) | 535 | 4.50 (0.03) | 4.30 (0.06) |
| **Sex** |  |  |  |  |  |  |  |
| Male | 807 (42.43%) | 807 | 7.51 (0.08) | 6.82 (0.17) | 801 | 4.55 (0.03) | 4.33 (0.06) |
| Female | 1095 (57.57%) | 1095 | 7.61 (0.07) | 7.15 (0.17) | 1085 | 4.48 (0.02) | 4.32 (0.06) |
| **Race/ethnicity** |  |  |  |  |  |  |  |
| White | 1793 (94.27%) | 1793 | 7.60 (0.06) | 7.40 (0.1) | 1777 | 4.52 (0.02) | 4.43 (0.04) |
| Black/African American | 31 (1.63%) | 31 | 6.32 (0.36) | 6.42 (0.34) | 31 | 4.07 (0.12) | 4.10 (0.12) |
| Asian/Pacific Islander | 22 (1.16%) | 22 | 7.39 (0.42) | 6.81 (0.4) | 22 | 4.47 (0.14) | 4.30 (0.14) |
| Hispanic | 29 (1.52%) | 29 | 7.57 (0.37) | 7.44 (0.35) | 29 | 4.46 (0.12) | 4.42 (0.12) |
| Other or multiracial | 27 (1.42%) | 27 | 6.87 (0.38) | 6.83 (0.36) | 27 | 4.40 (0.13) | 4.37 (0.13) |
| **Education** |  |  |  |  |  |  |  |
| High school or less | 423 (22.24%) | 423 | 7.38 (0.11) | 6.84 (0.18) | 419 | 4.45 (0.04) | 4.29 (0.06) |
| Some college | 635 (33.39%) | 635 | 7.33 (0.09) | 6.82 (0.17) | 627 | 4.45 (0.03) | 4.30 (0.06) |
| Bachelor’s degree | 435 (22.87%) | 435 | 7.89 (0.10) | 7.19 (0.19) | 433 | 4.59 (0.03) | 4.37 (0.07) |
| Graduate or professional degree | 409 (21.5%) | 409 | 7.72 (0.10) | 7.08 (0.19) | 407 | 4.55 (0.04) | 4.33 (0.07) |
| **Annual Household Income** |  |  |  |  |  |  |  |
| <$20,000 | 115 (6.05%) | 115 | 6.86 (0.19) | 6.82 (0.23) | 115 | 4.13 (0.06) | 4.13 (0.08) |
| $20,000-$49,999 | 341 (17.93%) | 341 | 7.27 (0.11) | 6.90 (0.18) | 339 | 4.36 (0.04) | 4.27 (0.06) |
| $50,000-$99,999 | 854 (44.90%) | 854 | 7.49 (0.08) | 6.97 (0.17) | 844 | 4.52 (0.03) | 4.40 (0.06) |
| ≥$100,000 | 592 (31.13%) | 592 | 7.93 (0.09) | 7.25 (0.18) | 588 | 4.64 (0.03) | 4.50 (0.06) |
| **Urban/rural residence** |  |  |  |  |  |  |  |
| Rural | 374 (19.66%) | 374 | 7.42 (0.11) | 6.85 (0.18) | 372 | 4.50 (0.04) | 4.31 (0.06) |
| Rural advantaged | 202 (10.62%) | 202 | 7.58 (0.15) | 7.06 (0.20) | 199 | 4.54 (0.05) | 4.36 (0.07) |
| Rural underserved | 67 (3.52%) | 67 | 7.51 (0.25) | 6.88 (0.27) | 67 | 4.52 (0.08) | 4.34 (0.10) |
| Urban | 247 (12.99%) | 247 | 7.32 (0.13) | 6.86 (0.19) | 245 | 4.53 (0.05) | 4.36 (0.07) |
| Urban advantaged | 957 (50.32%) | 957 | 7.69 (0.08) | 7.11 (0.16) | 950 | 4.51 (0.03) | 4.30 (0.06) |
| Urban underserved | 55 (2.89%) | 55 | 7.41 (0.27) | 7.13 (0.28) | 53 | 4.33 (0.09) | 4.29 (0.10) |
| **Treatment status** |  |  |  |  |  |  |  |
| Off treatment | 1077 (56.62%) | 1077 | 7.90 (0.07) | 7.30 (0.17) | 1070 | 4.52 (0.02) | 4.32 (0.06) |
| On treatment | 825 (43.38%) | 825 | 7.12 (0.08) | 6.67 (0.17) | 816 | 4.49 (0.03) | 4.33 (0.06) |
| **Most recent cancer diagnosis** |  |  |  |  |  |  |  |
| Breast | 369 (19.87%) | 369 | 7.43 (0.11) | 6.93 (0.18) | 365 | 4.55 (0.04) | 4.34 (0.06) |
| Hematological cancers^b^ | 78 (4.20%) | 78 | 7.15 (0.23) | 6.69 (0.26) | 74 | 4.41 (0.08) | 4.22 (0.09) |
| Female reproductive cancers^c^ | 105 (5.65%) | 105 | 7.07 (0.20) | 6.75 (0.23) | 104 | 4.36 (0.07) | 4.22 (0.08) |
| Prostate | 241 (12.98%) | 241 | 7.86 (0.14) | 7.18 (0.20) | 241 | 4.51 (0.05) | 4.25 (0.07) |
| Lung | 256 (13.79%) | 256 | 7.81 (0.13) | 7.25 (0.21) | 255 | 4.52 (0.04) | 4.37 (0.07) |
| Colorectal | 363 (19.55%) | 363 | 7.27 (0.11) | 6.84 (0.18) | 363 | 4.58 (0.04) | 4.39 (0.06) |
| Other^d^ | 445 (23.96%) | 445 | 7.92 (0.10) | 7.60 (0.19) | 443 | 4.49 (0.03) | 4.32 (0.07) |
| **Years since cancer diagnosis** |  |  |  |  |  |  |  |
| < 1 | 353 (18.56%) | 353 | 7.27 (0.11) | 6.72 (0.18) | 350 | 4.50 (0.04) | 4.33 (0.06) |
| 1-1.99 | 1061 (55.78%) | 1061 | 7.58 (0.07) | 6.97 (0.17) | 1054 | 4.51 (0.03) | 4.33 (0.06) |
| 2-2.99 | 304 (15.98%) | 304 | 7.80 (0.12) | 7.15 (0.19) | 301 | 4.54 (0.04) | 4.34 (0.07) |
| ≥ 3 | 184 (9.67%) | 184 | 7.66 (0.15) | 7.09 (0.20) | 181 | 4.45 (0.05) | 4.30 (0.07) |
| ^a^ Model 1 is not adjusted for covariates. Model 2 is adjusted for age at survey completion, race, sex, education, income, urban/rural advantage, treatment status, month of survey completion, years since initial cancer diagnosis, history of depression or anxiety, and the impact of COVID-19 on depression.  ^b^ Hematologic cancer include leukemia and lymphoma.  ^c^ Female reproductive cancers include vulvar, vaginal, cervical, endometrial, uterine, ovarian, and fallopian.  ^d^ Other cancer types with less than 50 survey responses including kidney, melanoma, and others.  Abbreviation: SE – standard error | | | | | | | |

| **Supplementary Table S2:** Mean self-efficacy by whether participants experienced interruptions to cancer-related healthcare during the COVID-19 pandemic (N = 1898) | | | | | | |
| --- | --- | --- | --- | --- | --- | --- |
|  | **Mean self-efficacy for**  **managing cancer** | | | **Mean self-efficacy for managing social interactions** | | |
|  | **N** | **Mean (SE)**  **Model 1**^a^ | **Mean (SE)**  **Model 2**^a^ | **N** | **Mean (SE)**  **Model 1**^a^ | **Mean (SE)**  **Model 2**^a^ |
| **Overall negative impacts of COVID-19 on cancer care**^b^ |  |  |  |  |  |  |
| No | 1169 | 7.85 (0.07) | 7.09 (0.17) | 1161 | 4.60 (0.02) | 4.40 (0.06) |
| Yes | 682 | 7.14 (0.08) | 6.62 (0.17) | 676 | 4.39 (0.03) | 4.29 (0.06) |
| *P-value*^c^ |  | *< .001* | *< .001* |  | *< .001* | *< .001* |
| **Self-reported problems getting specific prescription medications because of COVID-19** |  |  |  |  |  |  |
| No | 1818 | 7.62 (0.06) | 6.99 (0.16) | 1805 | 4.53 (0.02) | 4.34 (0.06) |
| Yes | 67 | 6.51 (0.24) | 6.31 (0.27) | 66 | 4.20 (0.08) | 4.12 (0.09) |
| *P-value*^c^ |  | *< .001* | *.003* |  | *< .001* | *.006* |
| **Self-reported a delay in cancer care for because of COVID-19** |  |  |  |  |  |  |
| No | 1330 | 7.72 (0.07) | 7.06 (0.17) | 1319 | 4.56 (0.02) | 4.42 (0.06) |
| Yes | 522 | 7.30 (0.09) | 6.74 (0.18) | 518 | 4.41 (0.03) | 4.28 (0.06) |
| *P-value*^c^ |  | *< .001* | *< .001* |  | *< .001* | *.008* |
| **COVID-19 negatively impacted ability to make decisions about healthcare** |  |  |  |  |  |  |
| 1 (Not true of me at all) | 1218 | 8.07 (0.07) | 7.30 (0.16) | 1210 | 4.65 (0.02) | 4.42 (0.06) |
| 2-3 | 419 | 7.07 (0.10) | 6.58 (0.18) | 417 | 4.37 (0.03) | 4.18 (0.06) |
| 4-5 | 155 | 6.44 (0.15) | 6.19 (0.21) | 154 | 4.30 (0.05) | 4.15 (0.08) |
| 6-7 (Very true of me) | 92 | 6.36 (0.20) | 6.19 (0.24) | 89 | 4.09 (0.07) | 4.18 (0.09) |
| *P-value*^c^ |  | *< .001* | *< .001* |  | *< .001* | *< .001* |
| **COVID-19 negatively impacted ability to obtain cancer care** |  |  |  |  |  |  |
| 1 (Not true of me at all) | 1357 | 7.94 (0.07) | 7.25 (0.16) | 1347 | 4.62 (0.02) | 4.43 (0.06) |
| 2-3 | 320 | 6.97 (0.11) | 6.60 (0.18) | 317 | 4.31 (0.04) | 4.21 (0.06) |
| 4-5 | 125 | 6.38 (0.18) | 6.13 (0.22) | 124 | 4.23 (0.06) | 4.21 (0.07) |
| 6-7 (Very true of me) | 83 | 6.54 (0.21) | 6.25 (0.25) | 83 | 4.26 (0.07) | 4.06 (0.08) |
| *P-value*^c^ |  | *< .001* | *< .001* |  | *< .001* | *< .001* |
| ^a^ Estimates adjusted for age at survey completion, race, sex, education, income, urban/rural advantage, treatment status, month of survey completion, years since initial cancer diagnosis, history of depression or anxiety, and the impact of COVID-19 on depression.  ^b^ Participant responded ‘yes’ to experiencing delays in receiving cancer-related care or problems receiving essential prescription medications, or expressed agreement (score ≥ 4 out of 7) with the statement “The COVID-19 epidemic has negatively impacted my ability to make decisions about my cancer care” or “The COVID-19 epidemic has negatively impacted my ability to obtain cancer care”.  ^c^ P-values represent a test of the null hypothesis that the mean values are equal across all groups.  Abbreviation: SE – standard error | | | | | | |

| **Supplementary Table S3:** Odds ratios (with 95% confidence intervals) for clinically significant distress and poor/fair general health for participants who experienced a disruption in cancer-related care due to COVID-19, and by level of self-efficacy (N = 1,897) | | | | | | |
| --- | --- | --- | --- | --- | --- | --- |
|  | **Significant distress**^a^ | | | **Poor/fair general health** | | |
|  | **Yes/total** | **OR**  **(95% CI)**  **Model 1**^b^ | **OR**  **(95% CI)**  **Model 2**^b^ | **Yes/total** | **OR**  **(95% CI)**  **Model 1**^b^ | **OR**  **(95% CI)**  **Model 2**^b^ |
| **Overall negative impacts of COVID-19 on cancer care**^c^ |  |  |  |  |  |  |
| No | 421/1161 | 1 (ref) | 1 (ref) | 253/1153 | 1 (ref) | 1 (ref) |
| Yes | 369/672 | 2.07 (1.70-2.52) | 1.70 (1.36-2.14) | 183/665 | 1.37 (1.10-1.70) | 1.13 (0.89-1.44) |
| *P-value*^d^ |  | *< .001* | *< .001* |  | *.006* | *.31* |
| **Self-reported problems getting specific prescription medications because of COVID-19** |  |  |  |  |  |  |
| No | 762/1801 | 1 (ref) | 1 (ref) | 414/1785 | 1 (ref) | 1 (ref) |
| Yes | 42/66 | 2.24 (1.34-3.76) | 1.84 (1.03-3.29) | 30/66 | 2.83 (1.72-4.67) | 2.12 (1.22-3.68) |
| *P-value*^d^ |  | *.002* | *.04* |  | *< .001* | *.007* |
| **Self-reported a delay in cancer care for because of COVID-19** |  |  |  |  |  |  |
| No | 521/1319 | 1 (ref) | 1 (ref) | 309/1309 | 1 (ref) | 1 (ref) |
| Yes | 265/514 | 1.57 (1.27-1.93) | 1.45 (1.14-1.85) | 123/507 | 1.05 (0.82-1.33) | 0.96 (0.74-1.24) |
| *P-value*^d^ |  | *< .001* | *.03* |  | *.70* | *.75* |
| **COVID-19 negatively impacted ability to make decisions about healthcare** |  |  |  |  |  |  |
| 1 (Not true of me at all) | 399/1208 | 1 (ref) | 1 (ref) | 225/1198 | 1 (ref) | 1 (ref) |
| 2-3 | 232/413 | 2.46 (1.95-3.10) | 1.66 (1.28-2.16) | 132/412 | 2.14 (1.65-2.76) | 1.86 (1.41-2.45) |
| 4-5 | 116/154 | 6.13 (4.16-9.04) | 3.63 (2.34-5.63) | 57/151 | 2.75 (1.92-3.96) | 2.00 (1.35-2.97) |
| 6-7 (Very true of me) | 54/91 | 2.85 (1.84-4.42) | 1.54 (0.90-2.64) | 30/90 | 2.19 (1.37-3.47) | 1.25 (0.74-2.10) |
| *P-value*^d^ |  | *< .001* | *< .001* |  | *< .001* | *< .001* |
| **COVID-19 negatively impacted ability to obtain cancer care** |  |  |  |  |  |  |
| 1 (Not true of me at all) | 484/1347 | 1 (ref) | 1 (ref) | 272/1337 | 1 (ref) | 1 (ref) |
| 2-3 | 188/315 | 2.47 (1.92-3.19) | 1.74 (1.30-2.32) | 101/313 | 1.98 (1.50-2.60) | 1.67 (1.24-2.24) |
| 4-5 | 83/124 | 3.38 (2.28-5.02) | 1.99 (1.26-3.12) | 47/121 | 2.56 (1.73-3.79) | 1.90 (1.24-2.91) |
| 6-7 (Very true of me) | 48/82 | 2.49 (1.57-3.92) | 1.75 (1.02-3.02) | 26/80 | 1.89 (1.16-3.08) | 1.31 (0.76-2.24) |
| *P-value*^d^ |  | *< .001* | < .001 |  | *< .001* | *< .001* |
| **General health** |  |  |  |  |  |  |
| Excellent | 25/11 | 1 (ref) | 1 (ref) | - | - | - |
| Very good | 161/548 | 1.57 (0.96-2.55) | 1.33 (0.77-2.29) | - | - | - |
| Good | 302/743 | 2.68 (1.67-4.30) | 1.97 (1.16-3.35) | - | - | - |
| Fair | 233/365 | 7.11 (4.31-11.73) | 4.35 (2.47-7.65) | - | - | - |
| Poor | 67/77 | 29.15 (12.98-65.45) | 19.43 (8.04-46.94) | - | - | - |
| *P-value*^d^ |  | *< .001* | *< .001* | *-* | *-* | *-* |
| **Mean self-efficacy for managing cancer^e^** |  |  |  |  |  |  |
| Q1 (1.0-6.3) | 333/469 | 1 (ref) | 1 (ref) | 263/461 | 1 (ref) | 1 (ref) |
| Q2 (6.4-7.8) | 220/431 | 0.42 (0.32-0.55) | 0.51 (0.37-0.69) | 102/430 | 0.23 (0.17-0.31) | 0.24 (0.18-0.33) |
| Q3 (8.0-9.0) | 153/499 | 0.18 (0.14-0.24) | 0.24 (0.17-0.33) | 57/494 | 0.10 (0.07-0.13) | 0.11 (0.08-0.16) |
| Q4 (9.2-10.0) | 102/481 | 0.11 (0.08-0.15) | 0.18 (0.13-0.26) | 26/481 | 0.04 (0.03-0.06) | 0.05 (0.03-0.09) |
| *P-value*^d^ |  | *< .001* | *< .001* |  | *< .001* | *< .001* |
| Continuous (per 1 unit increase) |  | 0.65 (0.61-0.68) | 0.71 (0.67-0.76) |  | 0.54 (0.51-0.58) | 0.57 (0.53-0.61) |
| **Mean self-efficacy for managing social interactions^e^** |  |  |  |  |  |  |
| Q1 (1.0-2.9) | 51/63 | 1 (ref) | 1 (ref) | 36/61 | 1 (ref) | 1 (ref) |
| Q2 (3.0-3.9) | 144/223 | 0.39 (0.19-0.77) | 0.34 (0.16-0.74) | 74/223 | 0.36 (0.20-0.64) | 0.36 (0.19-0.67) |
| Q3 (4.0-4.9) | 383/815 | 0.20 (0.10-0.37) | 0.24 (0.12-0.50) | 206/802 | 0.24 (0.14-0.41) | 0.32 (0.18-0.58) |
| Q4 (5) | 227/768 | 0.09 (0.05-0.17) | 0.14 (0.06-0.28) | 127/767 | 0.14 (0.08-0.24) | 0.23 (0.13-0.41) |
| *P-value*^d^ |  | *< .001* | *< .001* |  | *< .001* | *< .001* |
| Continuous (per 1 unit increase) |  | 0.39 (0.33-0.46) | 0.52 (0.44-0.63) |  | 0.53 (0.45-0.61) | 0.66 (0.56-0.78) |
| ^a^ Defined as score ≥ 4 out of 10 on National Comprehensive Cancer Network distress thermometer.  ^b^ Estimates adjusted for age at survey completion, race, sex, education, income, urban/rural advantage, treatment status, month of survey completion, years since initial cancer diagnosis, history of depression or anxiety, and the impact of COVID-19 on depression.  ^c^ Participant responded ‘yes’ to experiencing delays in receiving cancer-related care or problems receiving essential prescription medications, or expressed agreement (score ≥ 4 out of 7) with the statement “The COVID-19 epidemic has negatively impacted my ability to make decisions about my cancer care” or “The COVID-19 epidemic has negatively impacted my ability to obtain cancer care”.  ^d^ P-values represent a test of the null hypothesis that the mean values are equal across all groups.  ^e^ Higher scores reflect greater self-efficacy. | | | | | | |
